# Comparison of microbiomes of cold-water corals *Primnoa pacifica* and *Primnoa resedaeformis*, with possible link between microbiome composition and host genotype

**DOI:** 10.1038/s41598-018-30901-z

**Published:** 2018-08-17

**Authors:** Dawn B. Goldsmith, Christina A. Kellogg, Cheryl L. Morrison, Michael A. Gray, Robert P. Stone, Rhian G. Waller, Sandra D. Brooke, Steve W. Ross

**Affiliations:** 1 St. Petersburg Coastal and Marine Science Center, US Geological Survey, St. Petersburg, FL United States of America; 2 Leetown Science Center, US Geological Survey, Kearneysville, WV United States of America; 3 Auke Bay Laboratories, Alaska Fisheries Science Center, NOAA Fisheries, 17109 Point Lena Loop Road, Juneau, AK United States of America; 4 Darling Marine Center, University of Maine, Walpole, ME United States of America; 5 Coastal and Marine Laboratory, Florida State University, St. Teresa, FL United States of America; 6 Center for Marine Science, University of North Carolina at Wilmington, Wilmington, NC United States of America

## Abstract

Cold-water corals provide critical habitats for a multitude of marine species, but are understudied relative to tropical corals. *Primnoa pacifica* is a cold-water coral prevalent throughout Alaskan waters, while another species in the genus, *Primnoa resedaeformis*, is widely distributed in the Atlantic Ocean. This study examined the V4-V5 region of the 16S rRNA gene after amplifying and pyrosequencing bacterial DNA from samples of these species. Key differences between the two species’ microbiomes included a robust presence of bacteria belonging to the Chlamydiales order in most of the *P. pacifica* samples, whereas no more than 2% of any microbial community from *P. resedaeformis* comprised these bacteria. Microbiomes of *P. resedaeformis* exhibited higher diversity than those of *P. pacifica*, and the two species largely clustered separately in a principal coordinate analysis. Comparison of *P. resedaeformis* microbiomes from samples collected in two submarine canyons revealed a significant difference between locations. This finding mirrored significant genetic differences among the *P. resedaeformis* from the two canyons based upon population genetic analysis of microsatellite loci. This study presents the first report of microbiomes associated with these two coral species.

## Introduction

Tropical corals provide critical habitat for a vast number of marine species^1^, and recent expansion of deep-water marine research has revealed that cold-water coral ecosystems are abundant and equally critical to marine biodiversity^2^. Deep-sea and other cold-water corals are habitats for a wide variety of animals, including dozens of fish species^3–6^. Cold-water corals are also critical habitats for thousands of invertebrate species^7^. The biodiversity associated with cold-water corals continues to increase when microscopic associates are taken into account. All corals host rich microbial communities, and the microbiomes of cold-water corals are increasingly the focus of investigation into the complex interactions and symbioses among the members of the coral holobiont. Much of the research into the microbiomes of cold-water corals has centered on *Lophelia pertusa* and other stony corals^8–17^. However, deep-sea gorgonian coral microbiomes are beginning to be investigated^18–20^.

Only a few cold-water gorgonian coral genera have been the subject of microbiome comparisons between species. Microbiomes of two temperate *Muricea* species (*M. californica* and *M. fruticosa*) from the kelp forests of southern California were recently compared, revealing that the two species harbored distinct microbial communities^21^. A survey of temperate Mediterranean *Eunicella* species (*E. singularis*, *E. cavolini*, and *E. verrucosa*) found each species had a unique core microbiome. However, there was overlap of shared sequences, particularly *Endozoicomonas*, between the species^22^. In contrast, two deep-sea *Anthothela* species from the western Atlantic Ocean had essentially indistinguishable bacterial communities^20^. Further, samples of the two *Anthothela* species were collected in two different submarine canyons, but their geographic origin had no impact on the microbiome composition.

In this study, two species of *Primnoa* corals were sampled for the purpose of characterizing and comparing their microbial communities. The corals were sampled in two different ocean basins: *P. pacifica* samples were collected from one location in the Gulf of Alaska in the Pacific Ocean, and *P. resedaeformis* samples were collected from two submarine canyons in the North Atlantic Ocean, approximately 140 km apart. Collection of two different species enabled us to compare their microbiomes and determine which microbes were core to the genus and which were core to each species. In light of reports showing that cold-water corals have conserved bacterial communities^13,20^, we hypothesized that the microbiomes of *Primnoa* corals contain core bacterial species common to all members of the genus. We further hypothesized that *P. resedaeformis* and *P. pacifica* contain bacteria common to each coral species but not part of the core genus microbiome.

Collection of *P. resedaeformis* from separate submarine canyons allowed us to examine whether corals of the same species in different locations contained distinctive microbial communities. We expected the *P. resedaeformis* populations to have similar microbiomes based on a study of *Anthothela* coral microbiomes from the same locations^20^. When significant microbiome variation between canyons was detected, we analyzed microsatellite loci of the *P. resedaeformis* samples to examine whether host population genetics could be an underlying factor.

## Results

Six samples of *Primnoa pacifica* were collected from Tracy Arm Fjord in the Gulf of Alaska in 2011 and 2012, at depths ranging from approximately 10 to 16 m (Table 1). *Primnoa resedaeformis* samples were collected from Baltimore Canyon in 2012 (nine samples) and from Norfolk Canyon in 2013 (five samples) in the Atlantic Ocean at depths ranging from approximately 410 to 580 m (Norfolk) and 380 to 500 m (Baltimore) (Table 1). DNA was extracted from each sample as described in the Materials and Methods section. After amplification and sequencing of the 16S rRNA gene, sequence analysis was conducted in order to examine the microbial communities of each sample.

**Table 1:** *Primnoa* sample locations and environmental parameters.

Sample	Year	Collection Location	Ocean Basin	Temp (°C)	Depth (m)	Salinity (psu)
**PR_BC_01**	2012	Baltimore Canyon	Atlantic	6.2	450	35.1
**PR_BC_02**	2012	Baltimore Canyon	Atlantic	9.0	383	35.2
**PR_BC_03**	2012	Baltimore Canyon	Atlantic	7.4	443	35.1
**PR_BC_04**	2012	Baltimore Canyon	Atlantic	7.4	443	35.1
**PR_BC_05**	2012	Baltimore Canyon	Atlantic	7.5	430	35.0
**PR_BC_06**	2012	Baltimore Canyon	Atlantic	7.5	431	34.9
**PR_BC_07**	2012	Baltimore Canyon	Atlantic	7.3	506	35.1
**PR_BC_08**	2012	Baltimore Canyon	Atlantic	7.3	494	35.1
**PR_BC_09**	2012	Baltimore Canyon	Atlantic	7.6	500	35.1
PR_BC_10	2012	Baltimore Canyon	Atlantic	7.6	508	35.1
PR_NC_01	2012	Norfolk Canyon	Atlantic	6.2	535	35.0
PR_NC_02	2012	Norfolk Canyon	Atlantic	6.6	523	35.1
PR_NC_03	2012	Norfolk Canyon	Atlantic	6.3	434	35.0
**PR_NC_04**	2013	Norfolk Canyon	Atlantic	10.8	411	35.5
PR_NC_05	2013	Norfolk Canyon	Atlantic	9.0	441	35.2
**PR_NC_06**	2013	Norfolk Canyon	Atlantic	9.0	441	35.3
**PR_NC_07**	2013	Norfolk Canyon	Atlantic	6.3	498	35.0
**PR_NC_08**	2013	Norfolk Canyon	Atlantic	6.3	498	35.0
PR_NC_09	2013	Norfolk Canyon	Atlantic	6.6	479	35.1
**PR_NC_10**	2013	Norfolk Canyon	Atlantic	5.5	576	35.0
**PP_GA_01**	2012	Gulf of Alaska	Pacific	5.0	9.8	30.1
**PP_GA_02**	2012	Gulf of Alaska	Pacific	5.0	13.1	30.1
PP_GA_03	2012	Gulf of Alaska	Pacific	5.0	11.6	30.1
**PP_GA_04**	2012	Gulf of Alaska	Pacific	5.0	16.2	30.1
**PP_GA_05**	2011	Gulf of Alaska	Pacific	4.6	13.4	26.9
**PP_GA_06**	2011	Gulf of Alaska	Pacific	4.6	12.8	26.9
**PP_GA_07**	2011	Gulf of Alaska	Pacific	4.6	12.5	26.9

Samples beginning with “PR” are *P. resedaeformis*. Samples beginning with “PP” are *P. pacifica*. Date and time of collection and latitude and longitude for each sample are provided in Supplementary Table S2. Samples in bold were analyzed in this study.

### Bacterial diversity

Bacterial diversity of each sample was assessed through alpha diversity measurements including ACE richness (abundance-based coverage estimator), Chao1 richness, Shannon index, reciprocal Simpson index, and Simpson evenness index^23–26^ (Table 2). By every diversity measurement, the Atlantic *P. resedaeformis* samples exhibited higher average richness and evenness than the Pacific *P. pacifica* samples. The *P. resedaeformis* samples also exhibited a wider range of diversity measurements than the *P. pacifica* samples. Not only did the *P. resedaeformis* samples collectively have higher mean diversity (mean ACE richness 260.51, mean Chao1 richness 253.18, mean Shannon index 4.62, mean reciprocal Simpson index 9.70, mean Simpson evenness 0.05) than *P. pacifica* (mean ACE richness 125.31, mean Chao1 richness 117.76, mean Shannon index 1.87, mean reciprocal Simpson index 2.91, mean Simpson evenness 0.03), but they also included the samples with the highest individual diversity measurements. The samples with the highest values for ACE richness and Chao1 richness (sample PR_BC_02, with ACE richness of 448.65 and Chao1 richness of 414.56) as well as reciprocal Simpson index and Simpson evenness (sample PR_BC_09, with reciprocal Simpson index of 19.55 and Simpson evenness of 0.116) both came from Baltimore Canyon, while the sample with the highest Shannon index (which, like the Simpson index, incorporates richness and evenness^26,27^) came from Norfolk Canyon (sample PR_NC_06, with a Shannon index of 6.12). The least diverse samples came from the Gulf of Alaska.

**Table 2:** Alpha diversity associated with each *Primnoa* sample analyzed in this study. Samples from Baltimore Canyon and Norfolk Canyon are *P. resedaeformis*; samples from the Gulf of Alaska are *P. pacifica*.

Sample	Collection Location	Sequence Reads*	OTUs	ACE Richness	Chao1 Richness	Shannon Index	Reciprocal Simpson Index	Simpson Evenness
PR_BC_01	Baltimore Canyon	22,448	84	132.83	115.50	1.92	1.88	0.022
PR_BC_02	Baltimore Canyon	17,031	314	448.65	414.56	5.68	12.63	0.040
PR_BC_03	Baltimore Canyon	23,285	66	91.51	91.00	1.36	1.44	0.022
PR_BC_04	Baltimore Canyon	11,425	207	291.87	273.50	4.58	7.26	0.035
PR_BC_05	Baltimore Canyon	7,546	219	270.61	265.50	5.21	11.40	0.052
PR_BC_06	Baltimore Canyon	14,653	297	427.07	402.22	5.22	11.02	0.037
PR_BC_07	Baltimore Canyon	6,773	235	304.82	300.28	5.25	10.09	0.043
PR_BC_08	Baltimore Canyon	5,024	115	128.05	128.13	4.37	7.39	0.064
PR_BC_09	Baltimore Canyon	4,261	168	234.50	233.81	5.28	19.55	0.116
PR_NC_04	Norfolk Canyon	2,561	178	242.39	233.62	4.75	10.84	0.061
PR_NC_06	Norfolk Canyon	3,176	273	313.93	318.02	6.12	14.66	0.054
PR_NC_07	Norfolk Canyon	4,753	183	238.74	232.88	5.01	9.89	0.054
PR_NC_08	Norfolk Canyon	5,820	241	294.42	289.37	5.21	6.32	0.026
PR_NC_10	Norfolk Canyon	2,557	159	227.78	246.14	4.78	11.39	0.072
**Mean (standard deviation) for** ***P***. ***resedaeformis***				260.51 (102.0)	253.18 (95.5)	4.62 (1.3)	9.70 (4.7)	0.05 (0.025)
PP_GA_01	Gulf of Alaska	43,163	32	78.36	84.50	0.31	1.06	0.033
PP_GA_02	Gulf of Alaska	44,282	53	101.29	86.83	0.71	1.17	0.022
PP_GA_04	Gulf of Alaska	22,140	100	159.72	145.15	2.81	3.38	0.034
PP_GA_05	Gulf of Alaska	23,068	98	160.37	152.47	1.95	1.82	0.019
PP_GA_06	Gulf of Alaska	22,242	141	179.60	176.36	4.24	8.60	0.061
PP_GA_07	Gulf of Alaska	35,370	47	72.49	61.25	1.22	1.45	0.031
**Mean (standard deviation) for** ***P. pacifica***				125.31 (46.8)	117.76 (46.1)	1.87 (1.5)	2.91 (2.9)	0.03 (0.02)

*Samples were rarefied to 2,557 sequences before calculation of diversity metrics.

**Figure 1:**
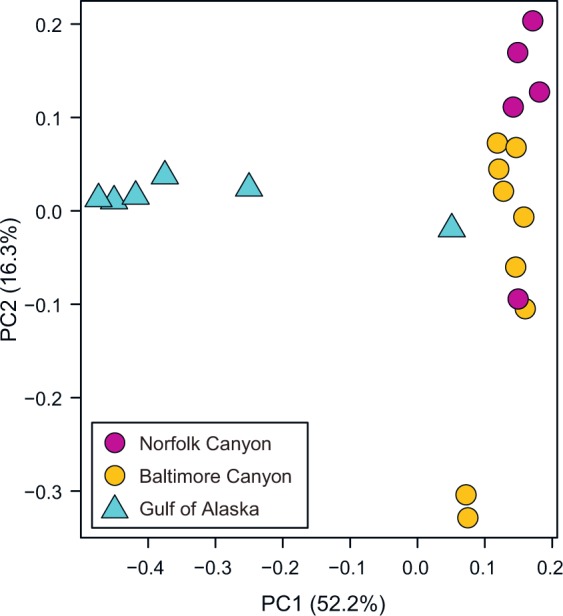
Principal coordinate analysis (PCoA) plot of weighted UniFrac distance. PCoA was used to plot beta diversity of coral-associated bacterial communities using the weighted UniFrac distance matrix.

Beta diversity measurements were visualized using principal coordinate analysis (PCoA). Similarity matrices were computed using four metrics: weighted and unweighted UniFrac (which incorporate phylogenetic distance), Bray-Curtis, and Sorensen-Dice. Analysis using the weighted UniFrac similarity matrix (Fig. 1) explained the greatest amount of variation among the samples. The metrics based on presence/absence (unweighted UniFrac and Sorensen-Dice) displayed greater separation between the two *Primnoa* species than the abundance-weighted metrics (weighted UniFrac and Bray-Curtis), and more overlap between the Norfolk Canyon and Baltimore Canyon *P. resedaeformis* samples. Analysis using the Bray-Curtis metric explained less of the variation among the samples, but revealed greater separation between *P. resedaeformis* samples from the two canyons.

### Bacterial community composition

**Figure 2:**
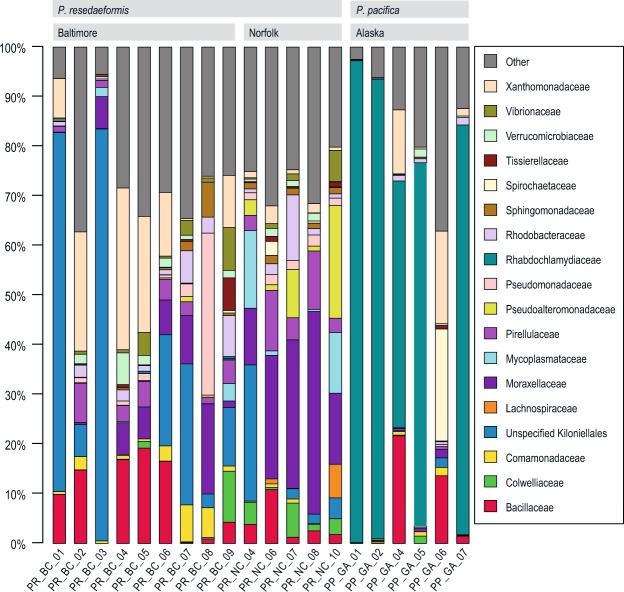
Relative abundance of bacterial families (or lowest identifiable phylogenetic level) in *Primnoa* samples. Bacterial groups shown present at ≥5% relative abundance in at least one sample. All remaining taxa are summarized under “Other”.

Analysis of the taxonomic makeup of their bacterial communities revealed differences between the two species of *Primnoa*. Bacteria from the Rhabdochlamydiaceae family (in the Chlamydiales order) dominated five of the six *P. pacifica* samples (Fig. 2). In those samples, Rhabdochlamydiaceae bacteria constituted from 49% to 97% of the bacterial communities, with three communities comprising more than 80% from that family. In contrast, no *P. resedaeformis* bacterial community consisted of more than 0.5% Rhabdochlamydiaceae. The *P. resedaeformis* samples instead had greater abundances of several families in the Proteobacteria phylum, including Xanthomonadaceae, Pseudomonadaceae, Pseudoalteromonadaceae, Moraxellaceae, and an unspecified Kiloniellales family (Fig. 2).

Of the families comprising at least 5% of any sample’s bacterial community, three families were absent from the *P. pacifica* samples, appearing only in *P. resedaeformis* samples (Lachnospiraceae, Mycoplasmataceae, and Pseudoaltermonadaceae), while three families appeared in every sample (Moraxellaceae, Pseudomonadaceae, and Rhodobacteraceae) (Fig. 2). However, the Moraxellaceae family played a larger role in the *P. resedaeformis* samples than in the *P. pacifica* samples. That family represented more than 5% of the bacterial community in all but three of the Canyon samples, but comprised less than 1% of every Alaska sample but one (in which it represented 1.7%) (Supplementary Table S1).

### ANOSIM and SIMPER analyses

Analysis of similarity (ANOSIM) between the *P. resedaeformis* and *P. pacifica* samples was conducted using the weighted UniFrac distance between each pair of samples (because that metric explained the greatest amount of variation among the samples). The ANOSIM R statistic was 0.839, showing a significantly high level of dissimilarity between the two species (p = 0.0001). Average similarity of each pair of samples within *P. resedaeformis* (SIMPER) was 31.65%. (Note that SIMPER analysis is based on Bray-Curtis dissimilarity, rather than weighted UniFrac distance, which means that the SIMPER analyses incorporate differences in abundance of OTUs but not phylogenetic distance between OTUs.) The largest contributors to that similarity were members of Kiloniellales (5.87%) and two *Acinetobacter* OTUs that totaled 5.64%. Within *P. pacifica*, average similarity of each pair of samples was 39.28%. The largest contributor to that similarity was the Chlamydiales OTU, at 37.54%. The next nearest contributor, an unassigned OTU, contributed 4.28%. The average dissimilarity between the two species was 83.32%. Again, the Chlamydiales OTU, the greatest contributor, was responsible for 7% of the dissimilarity, while Kiloniellales contributed 3.25%.

### Core microbiomes

**Figure 3:**
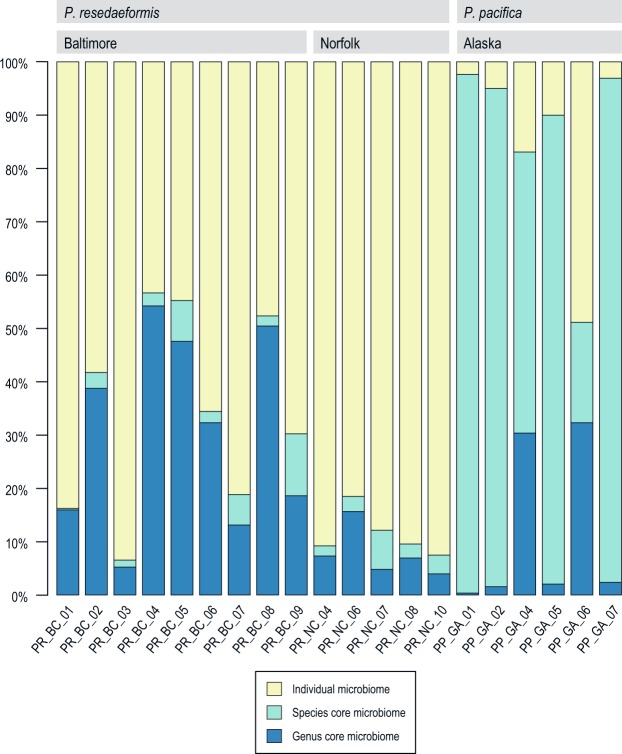
Relative abundance of genus core microbiome (OTUs found in all *Primnoa* samples), species core microbiome (OTUs found in all samples of *P. pacifica* or all samples of *P. resedaeformis*), and individual microbiome (remaining variable OTUs present in each coral colony).

The set of OTUs shared among all samples represents the *Primnoa* genus core microbiome (Fig. 3, Supplementary Table S1). Six OTUs appear in all *Primnoa* samples, from five microbial genera: two OTUs in the *Pseudomonas* genus, and one OTU each from the *Lysobacter*, *Bacillus*, *Acinetobacter*, and *Propionibacterium* genera. These *Primnoa* genus core OTUs comprise from 0.35% of the relative abundance in a *P. pacifica* sample to 54.2% in a *P. resedaeformis* sample from Baltimore Canyon (Fig. 3).

The species core microbiome for *P. resedaeformis* consists of four OTUs (Supplementary Table S1). Three OTUs were classified to the family level (Vibrionaceae, Pirellulaceae, and Rhodobacteraceae) and one to the genus level (*Sphingobium*). These OTUs make up 0.3% to 11.6% relative abundance of the *P. resedaeformis* samples. The species core microbiome for *P. pacifica* consisted of 19 OTUs (Supplementary Table S1), and constituted 18.8% to 97.3% of the bacterial community for the Pacific samples (Fig. 3). The largest contributor to the species core microbiome for *P. pacifica* was the Chlamydiales OTU. In four of the six samples, more than 74% of the entire microbiome comprised this OTU; in the other two samples, this OTU contributed 49.1% (PP_GA_04) and 0.013% (PP_GA_06). With one exception (PP_GA_06, the *P. pacifica* sample that is low in Rhabdochlamydiaceae), the individual microbiomes of the Pacific samples make up a much smaller portion of the entire microbiome than they do in the Atlantic samples.

### Inter-canyon comparison of *P. resedaeformis*

**Figure 4:**
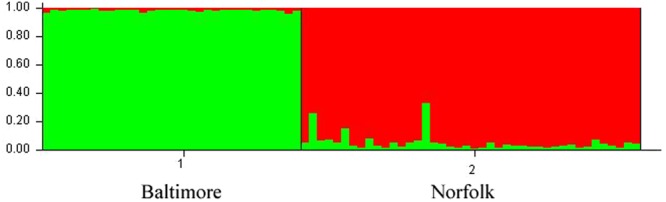
Bayesian clustering of *Primnoa resedaeformis* individuals from the Mid-Atlantic Bight canyons (Baltimore Canyon N = 32; Norfolk Canyon N = 42) based upon multilocus genotypes at eight microsatellite loci. Within bar plots, each *P. resedaeformis* individual is represented by a vertical bar partitioned into sections with lengths proportional to estimated probability of membership into K clusters, with the optimal number of clusters at K = 2.

Microsatellite profiling of the host populations in this study revealed a clear genetic difference between the two canyons. First, a Bayesian clustering analysis (STRUCTURE) recovered two distinct groupings based upon canyon of origin (Fig. 4). The genetic assignment of Norfolk Canyon *P. resedaeformis* samples suggest a few instances of mixed ancestry from Baltimore Canyon, which is concordant with larval transport following the slow southwestward flow of shelf and slope waters in the Mid-Atlantic Bight^28^. Second, a pairwise estimate of *F*_ST_ allele frequency-based measure of population differentiation^29^ between *P. resedaeformis* canyon populations was high and significant (*F*_ST_ = 0.117, p < 0.001). None of the loci appeared to be under selection based on the *F*_ST_-outlier test performed using LOSITAN^30^. Third, genetic assignment methods correctly assigned individuals to their canyon of origin at 97% and 93% to Baltimore and Norfolk Canyons, respectively. This high assignment success is only slightly less than the 100% correct assignment to species using a portion of the same microsatellite markers for *P. resedaeformis* and *P. pacifica*^31^.

#### ANOSIM/SIMPER

Additional ANOSIM and SIMPER analyses were conducted to analyze similarity between samples from Baltimore Canyon and Norfolk Canyon. The difference between bacterial communities from the two canyons was weaker (R = 0.254) than the difference between the two species, and the difference was barely significant (p = 0.047). However, the detection of a significant (albeit weak) difference between bacterial communities of the two *P. resedaeformis* populations is consistent with the microsatellite analysis that identified two distinct clusters of canyon samples (Fig. 4).

Considering each set of Canyon samples separately, the Norfolk Canyon samples had an average within-group similarity of 38.84%, while the Baltimore Canyon samples had an average within-group similarity of 34.65%. For Norfolk Canyon, the primary contributor to the similarity of the samples was *Acinetobacter*, which contributed (among two OTUs) nearly 12% of the average similarity. In Baltimore Canyon, the highest-contributing OTU was Kiloniellales (7.38%). Between the two canyons, the bacterial communities in *P. resedaeformis* were on average 72.35% dissimilar. Together, two *Acinetobacter* OTUs contributed just over 4% to this dissimilarity. Beyond that, no individual OTU contributed more than 3% to this dissimilarity. It is noteworthy that in contrast to the findings here, the microbiomes of the two *Anthothela* species studied in Lawler *et al*.^20^ exhibited no difference in bacterial community composition despite originating from the same two canyons sampled here.

#### Bacterial community composition

Differences emerged in the bacterial communities between the two submarine canyons. Two Norfolk Canyon samples contained moderate amounts of Mycoplasmataceae (12–15%), while Mycoplasmataceae did not exceed 3.6% of the bacterial community in any Baltimore Canyon sample (and were undetected in four samples) (Fig. 2). The Moraxellaceae family stood out because it was present in every Norfolk Canyon sample, ranging from 11% to 40% of those samples (Fig. 2). In contrast, only one Baltimore Canyon sample had a bacterial community that consisted of more than 10% Moraxellaceae (18% of sample PR_BC_08).

## Discussion

### Diversity in microbiomes of gorgonian corals

Temperate gorgonians in the Mediterranean have been shown to have very low diversity bacterial microbiomes, up to 90% composed of one or a few species, mainly of the genus *Endozoicomonas*^22,32^. It does not appear that deep-sea sibling species share this trait; for example, temperate *Paramuricea clavata* was dominated by *Endozoicomonas*^32^, but deep-sea *Paramuricea placomus* had no detectible *Endozoicomonas*, and no bacterial family represented more than 44% of the microbiome^19^. In this study, all but one of the *P. pacifica* samples (obtained from ca. 16 m) were dominated by Chlamydiae, much as Mediterranean gorgonian microbiomes are dominated by *Endozoicomonas*, whereas the *P. resedaeformis* samples obtained from the deep sea showed much more diversity (Fig. 2, Table 2). Because we were unable to sample at equivalent depths, we cannot separate differences based on species versus those due to depth or ocean basin, but mention this as a potential trend.

Unlike the comparison in this study, previous species comparisons within the same cold-water gorgonian genera have all been made using co-occurring species: *Muricea* spp. in California^21^, *Eunicella* spp. in the Mediterranean^22^, and *Anthothela* spp. in Atlantic submarine canyons^20^. The *Anthothela* microbiomes were not significantly different from each other. However, this may be due to the extremely low sequence divergence between the host species^20^. The three *Eunicella* species had significant overlap in their microbiomes, but this is largely due to the dominance of a single bacterial genus (*Endozoicomonas*), which kept the total diversity low. Even though *E. singularis* has zooxanthellae while the other two species (*E. cavolini* and *E. verrucosa*) do not, no obvious microbiome structuring was observed^22^. In contrast, the two *Muricea* species had very different microbiomes, and it was speculated that the presence of a photosymbiont in one species might be driving that difference^21^. Here, though both *Primnoa* species are azooxanthellate, they come from two different oceans, and it is interesting that the dissimilarity between the species is on par with that seen in co-occurring *Muricea* species.

### Comparing microbiomes of *P. resedaeformis* and *P. pacifica*

While the weighted UniFrac metric explains the greatest amount of variation among the samples (Fig. 1), the other beta diversity matrices provide additional insights. Both the Sorensen-Dice and unweighted UniFrac indices reveal a clear separation between *P. pacifica* and *P. resedaeformis*, indicating that the differences between the species’ microbiomes are being driven by the presence/absence of specific taxa (e.g., the Rhabdochlamydiaceae family in *P. pacifica*). The dominance of just one taxon in all but one of the *P. pacifica* samples’ microbiomes highlights the lower diversity of those samples reflected in the alpha diversity metrics. These two indices (Sorensen-Dice and unweighted UniFrac) also show overlap of the *P. resedaeformis* samples from different canyons. Further, the Bray-Curtis metric, which is based on OTUs, has no overlap between the canyon samples at all whereas weighted UniFrac, which factors in phylogenetic relatedness, shows one Norfolk Canyon sample clustering with the Baltimore Canyon samples (Fig. 1). In contrast, diversity matrices that factor in abundance of taxa as well as presence/absence (weighted UniFrac and Bray-Curtis) show much cleaner separation between the two canyon populations, indicating that it is not unique taxa that characterize the two canyon populations, but rather abundance ratios (e.g., higher relative abundance of Moraxellaceae in Norfolk samples; Fig. 2).

Though it is unclear why one *P. pacifica* sample (PP_GA_06) clustered with the *P. resedaeformis* samples (Fig. 1; Fig. 2), environmental conditions are unlikely to be the cause. The Alaskan coral was sampled at a temperature of 4.6 °C (versus 7.4 to 9 °C for the closest Atlantic samples), depth of 12.8 m (versus 383 to 500 m), and salinity of 26.9 psu (versus 35 psu). Given that the environmental conditions of the outlying *P. pacifica* sample were similar to those of the other Alaska samples, and dissimilar from those of the canyon samples, those conditions cannot account for its clustering with the canyon samples. However, it is not necessarily surprising that it clustered near the more diverse canyon samples, because its bacterial community was the most diverse of all the Alaska samples by every measure (Table 2).

### Core genus microbiome

The core genus microbiome consists of six OTUs, including *Propionibacterium*. The *Propionibacterium* OTU is noteworthy because the same OTU appears in the core microbiomes of two other cold-water corals: *Paramuricea placomus*^19^ and *Anthothela* sp.^20^. While the *Propionibacterium* OTU detected in *Primnoa* is two bases longer than the same OTU in *Paramuricea* and *Anthothela*, the three sequences are identical over the 331 bases they share. The similarity of the sequence of this persistent microbiome member among the three cold-water corals may be due to the close phylogenetic relationship among the three coral species; all fall within the Alcyonacea order. Not only is *Propionibacterium* a persistent member of the core microbiome of cold-water gorgonians, but it has also been identified as a rare but conserved member of the core microbiome of many stony tropical coral species^33–38^. However, its function in these microbiomes remains unknown^39^.

The other five core *Primnoa* OTUs (Supplementary Table S1) are also members of the *Paramuricea placomus* microbiome. The *Bacillus* and *Lysobacter* OTUs are identical to their counterparts found in *P. placomus*. The *Acinetobacter* OTU is two nucleotides longer in *Primnoa* than in *P. placomus*, but the OTUs are 99% identical over the 329 nucleotides they share. Comparing one set of core *Pseudomonas* OTUs (NCUR_OTU2 in *Primnoa* and OTU 4474944 in *P. placomus*) reveals that they are 99% identical over their shared 329 nucleotides. The other set of core *Pseudomonas* OTUs (OTU 4406538 in *Primnoa* and OTU 4478861 in *P. placomus*), both 331 nucleotides long, are 96% identical. The complete identity or high similarity of these core microbial OTUs in two different cold-water octocoral families (Plexauridae for *Paramuricea*, Primnoidae for *Primnoa*) allows us to speculate that these bacterial species may play a critical role in the functioning of the coral holobiont, at least for members of the Alcyonacea order.

The bacteria that are core to the *Primnoa* genus also appear in the microbiomes of many other corals, including tropical scleractinians. All five families that make up the *Primnoa* genus core have been documented in the microbiome of *Porites lutea*^36^. Three families (Pseudomonadaceae, Bacillaceae, and Moraxellaceae) are core members of the mucus microbiome of *Mussimilia hispida*^40^. In addition, Pseudomonadaceae are core members of the *Coelastrea aspera* microbiome^34^ and associate with numerous other tropical stony corals, including *Stylophora pistillata*^41^, *Pachyseris speciosa*^37^, *Fungia echinata*^42^, *Orbicella faveolata*^43^, *O. annularis*^43^, and *Astrangia poculata*^44^, whose range extends from tropical to temperate regions. This family of bacteria also populates the microbiomes of tropical soft corals^45,46^, as well as cold-water gorgonians^19^. Pseudomonads may assist their coral hosts in a broad range of metabolic functions^47^, which could account for their wide distribution in coral species. In particular, sulfur cycling genes have been identified in *Pseudomonas* species^48^, as well as genes implicated in the degradation of hydrocarbons, including crude oil^49,50^. Degradation of polycyclic aromatic hydrocarbons may also be performed by Xanthomonadaceae bacteria^51^, found as part of the core *Primnoa* microbiome as well as in the microbiome of *Porites lutea*^36^.

Moraxellaceae bacteria appear in the microbiomes of many of the same coral species as Pseudomonadaceae^37,41–43,52^. Like Pseudomonads, some members of the Moraxellaceae family can break down oil^17,53^, and may use that ability to recycle carbon for corals. Bacillaceae bacteria also populate the microbiomes of soft^19,54^ and stony^54^ corals, including tropical scleractinians^36,40,54^, and may benefit the corals by producing antimicrobial compounds that prevent host infection^54,55^.

Core species microbiome: *Primnoa pacifica*.

#### Significance of Chlamydiae sequences

Despite the dominance of Rhabdochlamydiaceae sequences in the *P. pacifica* samples, the sequences of DNA amplified from one of those samples in this study using Chlamydiales 23S primers did not match that family when queried against the GenBank nucleotide database. Rather, the top hits for those sequences were to *Simkania negevensis*, a member of the Simkaniaceae family in the Chlamydiales order. This suggests that Sanger sequencing did not capture the dominant Chlamydiales OTU present in the Alaska samples. However, there is some ambiguity about the lower-level classification of the OTU dominating the *P. pacifica* samples. Though Greengenes (within QIIME) identifies that OTU as a member of the *Rhabdochlamydia* genus (family Rhabdochlamydiaceae), the Ribosomal Database Project (RDP) Classifier^56^ identifies it as a member of the *Parachlamydia* genus in the Parachlamydiaceae family. It is not uncommon for databases to provide conflicting assignments at lower classification levels for a variety of reasons, including mislabeled sequences, errors in sequencing, and disproportionate population of databases by human-health-related bacteria rather than environmental samples^57^. Thus while we can confidently assign the OTU to the Chlamydiales order, database discrepancies prevent us from definitively classifying it to a family or genus.

We could find no reports of bacteria from the Rhabdochlamydiaceae family associated with corals or marine invertebrates. Chlamydiales sequences were recently found to be abundant in the microbiomes of cold-water sponges from the North Sea^58^. Relatively high percentages (>14%) of the bacterial communities associated with the sponges consisted of members of the Chlamydiales order. However, the sequences in that study that could be classified further were in the Parachlamydiaceae family, and are not closely related to the Chlamydiales sequences detected here. The only reports linking corals to Chlamydiales occur at the order, class, or phylum level, or through a histological study rather than a sequence-based study. Because none of the studies targeted the same region of the 16S gene as our study, we could not directly compare their sequences to ours. However, a *Chlamydia*-like bacteria was found in the microbiome of a shallow-water stony coral, *Isopora palifera*, comprising up to 33% of the bacterial communities in some samples off the coast of Taiwan^59^. At the phylum level, representatives of the Chlamydia phylum were reported in 2012 as 2% of the microbiome of a sample of *Pocillopora verrucosa*, a stony coral sampled in the Red Sea^60^. Members of the Chlamydiae class were also recently found in Red Sea *P. verrucosa* at abundances of nearly 25% of several samples’ microbiomes^41^. In the histological study, Work and Aeby^61^ examined an archive of coral tissue samples to search for cell-associated microbial aggregates. In their examination of 131 species and 36 genera of corals, they found that one species, *Acropora acuminata*, stained positive with a Gimenez stain, indicating the presence of chlamydia- or rickettsia-like bacteria.

Chlamydia in general are obligate intracellular bacteria, and have long been studied as animal/human pathogens. Now that their diverse presence in the environment as symbionts has been discovered^62^, we can speculate on what role they might play in a symbiosis. Chlamydiales bacteria may fulfill a metabolic function that *Primnoa* cannot themselves perform. Wagner and Horn^62^ suggest that the nucleotide transport proteins encoded by Chlamydia genomes may assist in exchanging bacterial ADP with host ATP. Alternately, these Chlamydiales sequences may reflect intracellular symbionts of amoebae that may be unrecognized members of these coral microbiomes^63^. In any case, given the relatively recent discovery of these bacteria in coral microbiomes, and the limited number of such reports, their role in coral-associated microbial communities is unknown.

#### Remaining members of core *P. pacifica* microbiome

Most remaining members of the core species microbiome for *P. pacifica* (Supplementary Table S1) have also been documented in other coral hosts’ microbiomes. Two families are also part of the *Primnoa* core genus microbiome (Pseudomonadaceae and Moraxellaceae, discussed above). Rhodobacteraceae are particularly widespread. Most recently, Rhodobacteraceae have been reported as core members of *Mussimilia hispida* mucus^64^, and appear in numerous other stony corals^11,35–37,43,44,63^. Many gorgonian corals also host Rhodobacteraceae^19,21,46,65,66^. This family’s presence in such a wide variety of coral hosts suggests that the bacteria serve a general function for the coral animal, but it has been hypothesized that their ability to oxidize thiosulfate as well as break down chitinous exoskeletons may provide those benefits to their coral hosts^14^.

Another member of the *P. pacifica* species core, Bradyrhizobiaceae, is commonly found in other coral microbiomes^19,35,36,46,63^, and may assist its hosts by fixing nitrogen^67–69^. Rhodocyclaceae bacteria, found in *Porites lutea*^36^, may degrade aromatic hydrocarbons^51,70^, while both Rhodocyclaceae and Comamonadaceae (found in soft, stony, temperate, and tropical corals^19,36,37,71^) have been linked to denitrification^72,73^. The SAR324 clade, a group of Deltaproteobacteria, are capable of a variety of metabolic pathways^74^ and thus could provide flexibility to their coral host. *Streptococcus* bacteria have been reported in other corals’ microbiomes^34,36,75^, but their function as part of the coral holobiont is not yet known. Both *Staphylococcus*^36,37,41,54,75^ and Enterobacteriaceae^11,19,36,37,46,76^ may act as opportunistic pathogens^14,77,78^ in the corals whose microbiomes they inhabit.

Core species microbiome: *P. resedaeformis*.

The core species microbiome for *P. resedaeformis* is much smaller than that of *P. pacifica*, containing only four members (families Rhodobacteraceae, Pirellulaceae, Vibrionaceae, and *Sphingobium* in the Sphingomonadaceae family). These bacteria commonly associate with corals. As noted above in discussion of the *P. pacifica* core species microbiome, members of the Rhodobacteraceae family are widespread in coral microbiomes, and may serve either general or particularized functions for the host. Pirellulaceae bacteria have been reported both in scleractinian^11,36,44^ and gorgonian^19,66^ corals, and have been hypothesized to play a role in the nitrogen cycle within the coral holobiont^19,20,79,80^.

In addition to appearing as core members of the *P. resedaeformis* microbiome, Vibrionaceae bacteria were recently identified as core members of the microbiome of stony coral *Cladocora caespitosa*^81^, and appear in the microbiomes of corals worldwide^19,21,22,35–37,41,42,45,54,63–65,71,76,82–85^. Though Vibrios are often associated with coral disease, their widespread association with healthy corals suggests an important function in the normal functioning of the coral holobiont. Vibrionaceae bacteria may fix nitrogen for the host^68,86^, and may also produce biosurfactant to degrade oil^87^. *Sphingobium* species also degrade polycyclic aromatic hydrocarbons^88^, and members of the Sphingomonadaceae family have recently been documented in other coral hosts^11,36,37,44^.

### Inter-canyon comparison of *P. resedaeformis* populations

#### Microbiome diversity and host influence

A study of the soft coral *Lobophytum pauciflorum*’s microbiome found a higher abundance of Spirochaetes- and Rhodobacteraceae-related sequences in male versus female corals^89^. We did not see evidence of this pattern. However, we were only able to determine sex for 11 out of 20 samples, and only two of those were male (Supplementary Table S2). It has been suggested that microbiome composition and specificity may be influenced by the reproductive mode of the host coral, linking selection of specific bacterial groups to the host’s life history^41^. In a study of two tropical corals, the species that spawned (and therefore acquired its microbiome from the environment every time) always had the same species of *Endozoicomonas*, whereas the brooding species had a strong regional signal in its *Endozoicomonas* species, attributed to vertical transmission^41^. *Primnoa resedaeformis* is a broadcast spawner with external fertilization^90^, and thus any regional signal we observe in the microbiomes between the canyons cannot be attributed to host reproductive strategy.

Given the unique genetic signatures of *P. resedaeformis* in Baltimore and Norfolk Canyons, a possible explanation for the differences in microbiome composition detected between canyons may be the influence of host genotype on the microbiome. The possibility of coral host genotype affecting its bacterial associates has been proposed^11,91^ given that several studies have shown that coral microbiome or bacterial symbiont phylogenies correlated to host phylogeny^92,93^. Studies focused on the endangered tropical stony coral *Acropora cervicornis* found coral genotype to be linked to differences in thermal tolerance and bleaching^94,95^, growth rate^94^, and disease susceptibility^96,97^. Recent studies have also linked coral microbiome shifts to thermal tolerance^98^ and altered growth rates^99^, and there is an obvious link between the microbiome and disease susceptibility. Another recent soft coral study found that during stress experiments, the observed microbial profiles could be linked back to coral colonies, suggesting host genotype was driving their results^89^. Therefore, we suggest that the microbiome patterns in concert with the host population genetics observed between these two canyons may be another piece of evidence connecting coral microbiome selection to coral host genotype.

We cannot exclude the possibility that differences in the microbiomes between the two populations of canyon samples (*P. resedaeformis*) could stem from differences in environmental conditions under which the populations were sampled. However, the differences in the environmental conditions we measured were relatively minor (Table 1) and thus unlikely to be a factor in the differences in microbiomes between the two populations. Seasonality may play a role, given that the Baltimore Canyon samples were collected in August 2012, while the Norfolk Canyon samples were collected in May 2013 (Table S2). Differential abundance of several bacterial OTUs varied seasonally in the microbiomes of Mediterranean gorgonian corals (three *Eunicella* species and one *Leptogorgia* species), though no pattern to the variation could be detected^76^. The microbiome of temperate gorgonian *Paramuricea clavata* also varied with the season^100^. Seasonal shifts in relative bacterial abundance have also been observed in microbiomes associated with numerous non-gorgonian corals^44,59,71,101–103^. Note, however, that *Anthothela* species collected from the same two canyons at the same time points as the *P. resedaeformis* collections did not display any difference in their microbiomes^20,104^.

Moreover, it is possible that diet differs between the two populations of *P. resedaeformis*, given that there are differences in turbidity between the two canyons, which may affect the corals’ nutrition. While Baltimore Canyon has a persistent thick nepheloid layer^105^, turbidity layers in Norfolk Canyon are thinner and more uniform^106^. In addition, seasonal changes in the composition of particulate matter have been documented in Baltimore Canyon^105^, indicating that coral diet could change seasonally. However, two populations of *Anthothela* species sampled in the same canyons contain nearly identical microbiomes^20^, and we can find no information to suggest that *Anthothela* and *Primnoa* corals have different diets. Nonetheless, the observed differences in the microbiomes of *P. resedaeformis* between the two canyons may result from differences in diet, seasonality, host genotype, or a combination of those factors.

## Conclusion

This study provides the first report of the microbial community associated with two *Primnoa* species of cold-water coral: *P. pacifica* from shallow water in Gulf of Alaska fjords, and *P. resedaeformis* from two submarine canyons in the western North Atlantic Ocean. One of the prominent differences between the two species’ microbiomes is the abundance of Chlamydiales bacteria in *P. pacifica*. The *P. resedaeformis* samples showed higher average mean diversity than those of *P. pacifica*. While five taxa (six OTUs) were core microbiome members conserved within the genus, principal coordinate analysis and ANOSIM statistics demonstrated that the two species’ microbiomes were relatively distinct. Within *P. resedaeformis*, a subtle but significant difference in bacterial communities was detectable between two submarine canyons. This pattern mirrors population genetic isolation demonstrated between the host coral population using microsatellite markers, raising the possibility that host genotype may play a role in differences in microbiomes between corals of the same species.

## Materials and Methods

### Sample sites and collection

*Primnoa pacifica* samples from Tracy Arm Fjord, Gulf of Alaska (Pacific Ocean) were collected at depths of 9.8 m to 16.2 m using SCUBA during research cruises in September 2011 and January 2012. Divers wearing clean nitrile gloves donned at the surface sampled the first *P. pacifica* colony located on the dive (i.e., no other corals were touched before collecting each of these samples). Small pieces of coral were broken from the colony by hand and placed into sterile 50-mL tubes (Fisher Scientific, Pittsburgh, PA). Environmental parameters were recorded for all samples (Table 1, Supplementary Table S2). Upon return to the surface, the ambient seawater in the tubes was decanted and replaced with RNAlater solution (Ambion, Waltham, MA). The tubes were kept at 4 °C overnight and then moved to −20 °C for long-term storage.

*Primnoa resedaeformis* samples were collected from Baltimore Canyon in the western North Atlantic Ocean (Table 1; Supplementary Table S2) on the NOAA ship *Nancy Foster* at depths of 383 m to 508 m using the remotely operated vehicle (ROV) *Kraken II* (National Undersea Research Technology and Education Center, University of Connecticut). Small pieces of colonies were collected using the ROV’s manipulator arm and placed into PVC tubes that had been washed, sterilized with ethanol, and filled with freshwater while the ROV was on deck. Care was taken to isolate samples from different coral colonies by placing only one sample per tube. Upon recovery of the ROV, the samples were removed from the tubes using ethanol-sterilized forceps, trimmed if necessary with ethanol-sterilized shears, and placed into 50-mL tubes. The tubes were filled with RNAlater and placed at 4 °C overnight, then transferred to −20 °C for long term storage. Georeferenced *P. resedaeformis* genetics samples were also stored in PVC tubes on the ROV. DNA from these samples was stabilized in 95% ethanol and Whatman FTA Technology Classic cards (GE Healthcare Bio-Sciences, Pittsburgh, PA). Additional *P. resedaeformis* samples were collected from Norfolk Canyon in the western North Atlantic Ocean on the NOAA ship *Ronald H. Brown* at depths of 411 m to 576 m using the ROV *Jason II* (Deep Submergence Laboratory, Woods Hole Oceanographic Institution) as described for the Baltimore Canyon samples.

### Sex determination

Coral samples collected for sex determination were placed into 10% fully buffered formalin during the cruise and then transferred into 70% ethanol in the laboratory. Sex of the coral samples was determined by histological examination of oocytes and spermatocysts as described in Mercier and Hamel^90^. Briefly, the calcified skeleton of each sample was dissolved in 10% hydrochloric acid, then rinsed in distilled water and dehydrated through a series of ethanol baths. The tissues were then cleared, embedded into paraffin, and sliced into 8-µm sections. After mounting and staining, images of the sections were taken using a digital camera attached to a compound microscope.

#### Nucleic acid extraction and 16S rRNA gene sequencing

DNA was extracted from the corals using the MoBio PowerPlant DNA Isolation Kit (Carlsbad, CA) according to the manufacturer’s instructions as modified by Sunagawa *et al*.^92,107^. Extractions were performed in triplicate and then pooled for each sample. Extracted DNA was quantified using the PicoGreen DNA quantification kit (Invitrogen, Grand Island, NY). For microsatellite genotyping, total DNA was isolated from preserved coral tissue and/or FTA card hole-punches using the Gentra PureGene Tissue kit (QIAGEN, Germantown, MD). The DNA was eluted in 30 µL of molecular grade water. DNA extracted from the coral samples was amplified using primers 563F and 926 R, which target the V4-V5 region of the 16S rRNA gene: forward primer 5′-AYTGGGYDTAAAGNG, reverse primer 5′-CCGTCAATTYYTTTRAGTTT^108^. The DNA was then sequenced by 454 pyrosequencing using GS FLX Titanium chemistry following Roche 454′s standard protocol for amplicons. Twenty samples were sequenced on each plate, so barcodes were attached to the primers before amplification (Integrated DNA Technologies, Inc., Coralville, IA). Amplification and 454 pyrosequencing were conducted by Selah Genomics (Greenville, SC).

### Bioinformatics and statistical analyses

Bioinformatic analysis of the sequences was performed using QIIME version 1.9.1^109^. A total of 1,517,466 raw reads were generated from 27 individual coral samples. Sequences were screened based on the following quality parameters: sequence length between 200 and 700 bp, minimum average quality score of 25, minimum quality score window of 50, maximum of one primer mismatch, maximum of six ambiguous bases, and a maximum six-homopolymer run^110^. Next, the sequences were denoised^110,111^. After screening and denoising, 409,886 sequences remained. Next, samples with a low yield of sequences were removed from the analysis. One sample (PR_BC_10) did not produce any reads, while three other samples (PR_NC_01, PR_NC_05, and PR_NC_09) each produced fewer than 2,500 reads. After removal of these samples, 23 samples and 404,999 sequences remained.

An open-reference method with a 97% similarity threshold^112^ was used to select OTUs in order to avoid discarding sequences that were not a perfect match to the Greengenes reference database release 13_8^113,114^. Chimeras were removed and the OTUs picked using usearch61^115^. Alignment was done with PyNAST (version 1.2.2)^116^. Representative sequences from each OTU were selected, assigned a taxonomic classification using uclust^115^, and used to generate a phylogenetic tree^117^. Non-bacterial sequences (i.e., Eukarya, Archaea, chloroplast, and mitochondria) and absolute singletons (defined as OTUs present only once in the analysis) were removed. After these steps, three samples had less than 2,500 sequences and were removed from the analysis (PR_NC_02, PR_NC_03, and PP_GA_03). Twenty samples and 321,578 sequences remained, with each individual sample library containing over 2,500 sequences. Samples were randomly rarefied to the size of the smallest remaining library (2,557 sequences) before diversity metrics were calculated^118^. A comprehensive list of scripts and parameters used in this analysis is presented as part of a USGS data release^119^ (https://doi.org/10.5066/F7P55KMJ).

Alpha and beta diversity measurements and OTU relative abundances were calculated using QIIME v. 1.9.1^109^. Beta diversity metrics were visualized via principal coordinate analysis (PCoA) using the vegan package^120^ in R^121^. PRIMER-E^122^ was used to calculate analysis of similarities (ANOSIM) from the weighted UniFrac distance matrix using 9999 permutations. PRIMER-E was also used to conduct SIMPER analysis, which quantifies within-group similarities and between-group dissimilarities, and identifies the OTUs that are responsible for each. Because SIMPER analysis is based on Bray-Curtis dissimilarity, the analysis incorporates differences in abundance of OTUs but not phylogenetic distance between OTUs. Relative abundance column graphs were prepared in R^121^. The core microbiome (set of shared OTUs) was derived from the OTU table prepared by QIIME before the rarefaction step. To be considered a member of the core genus microbiome, an OTU had to be present in 100% of samples. OTUs were designated as members of core species microbiomes when they were present in 100% of that species’ samples. The core microbiomes were analyzed in Excel and visualized with R.

### 23S Chlamydiales gene from *P. pacifica*

Because the QIIME analysis assigned some sequences from the *P. pacifica* samples to the *Rhabdochlamydia* genus (family Rhabdochlamydiaceae, order Chlamydiales), we attempted to amplify the 23S rRNA gene specific to the Chlamydiales order from bacterial DNA extracted from those samples. Primers U23F (5′-GATGCCTTGGCATTGATAGGCGATGAAGGA) and 23SIGR (5′-TGGCTCATCATGCAAAAGGCA)^123^ were used to amplify a product of approximately 600 bp. The 50-µL reaction mixture contained 25 µL AmpliTaq Gold 360 Master Mix (Applied Biosystems, Foster City, CA), 0.2 µM concentration of each primer, and 1 µL of template. Reaction conditions of Everett *et al*.^123^ were followed after 15 min of initial denaturation at 95 °C. One Alaska sample, PP_GA_01, yielded a visible PCR product. The product was cloned into the pDrive vector using the PCR Cloning Plus kit (QIAGEN, Germantown, MD) and used to transform competent cells. Inserts in positive transformants were sequenced by Eurofins Genomics (Louisville, KY). Vector sequences were identified with VecScreen^124^ and trimmed using EnzymeX v.3.3.3 (Nucleobytes, Amsterdam, The Netherlands). Sequences were aligned with Clustal Omega as implemented by EMBL-EBI^125,126^, revealing two variations of the sequences. The sequences were analyzed using BLASTN against the nt database^127^.

### Microsatellite genotyping and analyses

Next-generation sequencing was used to develop microsatellite loci for both *Primnoa* species sampled in this study^31^. Eight of these loci amplified successfully and were polymorphic in the Atlantic Canyons samples: Prim014, Prim026, Prim060, Prim068, Prim069, Prim074, Prim094, and Prim096. Loci were amplified singly via PCR following conditions in Morrison *et al*.^31^, with a final reaction volume of 20 µL.

In order to describe genetic relationships between populations, an allele frequency model-based Bayesian clustering approach^128^ was implemented in STRUCTURE v. 2.3.4^129^. This method infers the number of genetic clusters (*K*) from multi-locus genotype data by minimizing Hardy-Weinberg equilibrium and linkage disequilibrium among loci within groups and assigning individuals (probabilistically) to each cluster. Models utilizing collection location information to inform prior probability that an individual sample comes from a particular population have proven useful when detecting genetic structure in small datasets or when structuring is weak^129^. Therefore, we included sample location information to weight the model in favor of outcomes that are correlated with sampling information. Settings for all runs also included an admixture model (i.e., individuals may have mixed ancestry), correlated allele frequencies^130^, and 200,000 Markov chain Monte Carlo iterations after a burn-in of 50,000 iterations. Ten independent chains were run to test each value of *K*. The optimum number of clusters was determined by evaluating the values of *K* as the highest mean likelihood of the probability of the number of clusters given the data observed, ln Pr(X|*K*)^128^, and Δ*K*^131^. This information was compiled and graphed using STRUCTURE Harvester v.0.56.1^132^. Additionally, population genetic differentiation among canyons was examined through a pairwise *F*_ST_ allele frequency-based estimate^29^. Significance of the *F*_ST_ value was tested using 999 pairwise population permutations in GenAlEx v. 6.5b4^133,134^. Assignment tests were performed in GenAlEx using the leave-one-out procedure. Microsatellite loci were searched to identify those potentially under selection using LOSITAN (Antao *et al*.^30^), which implements *F*_ST_-outlier tests. Simulations were run under infinite alleles and stepwise mutation models using default settings (50,000 replications).

## Electronic supplementary material

Supplementary Material

Table S1

## Data Availability

The raw data files associated with the 16 S sequences in this study have been submitted to the NCBI Sequence Read Archive under Bioproject number PRJNA348705 and are also available from the USGS data release^119^ (https://doi.org/10.5066/F7P55KMJ). The bar codes used in sequencing can be found in mapping files, which are part of the data release. The USGS data release also contains the bioinformatics workflow, including scripts and parameters for each step. The 23 S sequences have been submitted to GenBank and assigned accession numbers KY010287 and KY010288; they are also part of the USGS data release. A separate USGS data release^135^ presents the microsatellite genotypes for *Primnoa resedaeformis* (https://doi.org/10.5066/F7B27T74).
