# Supplementary material for: Comparison of microbiomes of cold-water corals Primnoa pacifica and Primnoa resedaeformis, with possible link between microbiome composition and host genotype
Source: Sci Rep. 2018 Aug 17;8:12383. doi: 10.1038/s41598-018-30901-z (PMC6098105; doi:10.1038/s41598-018-30901-z)
Supplement: Supplementary file 1 — Supplementary Material [file 41598_2018_30901_MOESM1_ESM.docx]

**Supplementary Material**

**Comparison of microbiomes of cold-water corals *Primnoa pacifica* and *Primnoa resedaeformis*, with possible link between microbiome composition and host genotype**

Dawn B. Goldsmith^1^, Christina A. Kellogg^1*^, Cheryl L. Morrison^2^, Michael A. Gray^1^, Robert P. Stone^3^, Rhian G. Waller^4^, Sandra D. Brooke^5^, Steve W. Ross^6^

^1^St. Petersburg Coastal and Marine Science Center, US Geological Survey, St. Petersburg, FL, United States of America

^2^Leetown Science Center, US Geological Survey, Kearneysville, WV, United States of America

^3^Auke Bay Laboratories, Alaska Fisheries Science Center, NOAA Fisheries, 17109 Point Lena Loop Road, Juneau, AK, United States of America

^4^Darling Marine Center, University of Maine, Walpole, ME, United States of America

^5^Coastal and Marine Laboratory, Florida State University, St. Teresa, FL, United States of America

^6^Center for Marine Science, University of North Carolina at Wilmington, Wilmington, NC, United States of America

^*^Corresponding author: Christina A. Kellogg, St. Petersburg Coastal and Marine Science Center, US Geological Survey, 600 4th Street South, St. Petersburg, Florida 33701. Phone: (727) 502-8128. Fax: (727) 502-8181. Email: ckellogg@usgs.gov

**Table S1**. Abundance of all operational taxonomic units (OTUs) in *Primnoa* samples, along with taxonomic classification and representative sequence for each OTU. Shading of OTU identifier column indicates members of core microbiome for *Primnoa* genus (red), *P. pacifica* species (yellow), and *P. resedaeformis* species (blue).

[Table S1 is a separate document in Excel format.]

**Table S2**. Date, time, latitude and longitude of collection for each *Primnoa* sample, as well as gender when available. Samples beginning with “PR” are *P. resedaeformis*. Samples beginning with “PP” are *P. pacifica*. Time of collection indicates Eastern Daylight Time (EDT) which is 4 hours behind Coordinated Universal Time (UTC). Highlighted samples were analyzed in this study. NA: not available.

| **Sample Name**  **(this study)** | **Sample Name (original)** | **Date of Collection** | **Time of Collection** | **Latitude** | **Longitude** | **Gender** |
| --- | --- | --- | --- | --- | --- | --- |
| PR_BC_01 | NF12-1Q6 | 18 Aug 2012 | 17:25 | 38.149510 N | 73.837951 W | NA |
| PR_BC_02 | NF12-2Q6 | 19 Aug 2012 | 13:17 | 38.149088 N | 73.836225 W | F |
| PR_BC_03 | NF12-5Q6 | 23 Aug 2012 | 13:53 | 38.137820 N | 73.833683 W | M |
| PR_BC_04 | NF12-5Q7 | 23 Aug 2012 | 14:08 | 38.137777 N | 73.833598 W | NA |
| PR_BC_05 | NF12-6Q6 | 24 Aug 2012 | 12:12 | 38.139116 N | 73.833355 W | NA |
| PR_BC_06 | NF12-6Q7 | 24 Aug 2012 | 11:54 | 38.139081 N | 73.833381 W | NA |
| PR_BC_07 | NF12-9Q6 | 28 Aug 2012 | 13:07 | 38.151751 N | 73.839908 W | M |
| PR_BC_08 | NF12-9Q7 | 28 Aug 2012 | 13:28 | 38.151416 N | 73.839820 W | F |
| PR_BC_09 | NF12-10Q6 | 29 Aug 2012 | 12:30 | 38.166420 N | 73.855801 W | F |
| PR_BC_10 | NF12-10Q7 | 29 Aug 2012 | 12:07 | 38.167350 N | 73.854503 W | NA |
| PR_NC_01 | NF12-12Q6 | 5 Sept 2012 | 12:31 | 37.068361 N | 74.648271 W | NA |
| PR_NC_02 | NF12-12Q7 | 5 Sept 2012 | 12:55 | 37.067373 N | 74.648941 W | NA |
| PR_NC_03 | NF12-20Q1 | 13 Sept 2012 | 17:00 | 37.052256 N | 74.622626 W | NA |
| PR_NC_04 | RB684Q1 | 10 May 2013 | 10:42 | 37.068614 N | 74.644628 W | F |
| PR_NC_05 | RB684Q2 | 10 May 2013 | 12:08 | 37.067677 N | 74.643226 W | NA |
| PR_NC_06 | RB684Q3 | 10 May 2013 | 12:00 | 37.067687 N | 74.643226 W | F |
| PR_NC_07 | RB684Q4 | 10 May 2013 | 7:34 | 37.071599 N | 74.649173 W | NA |
| PR_NC_08 | RB684Q5 | 10 May 2013 | 7:40 | 37.071597 N | 74.649191 W | F |
| PR_NC_09 | RB686Q3 | 13 May 2013 | 11:23 | 37.058606 N | 74.605781 W | NA |
| PR_NC_10 | RB687Q2 | 14 May 2013 | 8:03 | 37.054961 N | 74.578277 W | NA |
| PP_GA_01 | AKPP1 | 4 Jan 2012 | NA | 57.888983 N | 133.31645 W | NA |
| PP_GA_02 | AKPP2 | 4 Jan 2012 | NA | 57.888983 N | 133.31645 W | NA |
| PP_GA_03 | AKPP3 | 5 Jan 2012 | NA | 57.888983 N | 133.31645 W | NA |
| PP_GA_04 | AKPP4 | 4 Jan 2012 | NA | 57.888983 N | 133.31645 W | F |
| PP_GA_05 | AKUT1 | 9 Sept 2011 | NA | 57.888983 N | 133.31645 W | NA |
| PP_GA_06 | AK325 | 9 Sept 2011 | NA | 57.888983 N | 133.31645 W | F |
| PP_GA_07 | AK342 | 9 Sept 2011 | NA | 57.888983 N | 133.31645 W | F |
